# Microneedles enhance the efficacy of topical dental anesthesia in male subjects: a randomized controlled trial

**DOI:** 10.1590/1807-3107bor-2026.vol40.023

**Published:** 2026-05-18

**Authors:** Stephany Di Carla Santos, Nádia Cristina Favaro Moreira, Henrique Ballassini Abdalla, Gabriela Gama Xavier Augusto, Yuri Martins Costa, Michelle Franz-Montan

**Affiliations:** (a) Universidade Estadual de Campinas – Unicamp, Piracicaba Dental School, Department of Biosciences, Piracicaba, SP, Brazil.; (b) Instituto de Pesquisas São Leopoldo Mandic, Faculdade São Leopoldo Mandic, Laboratory of Neuroimmune Interface of Pain Research, Campinas, SP, Brazil.

**Keywords:** Clinical Trial, Mouth, Administration, Topical

## Abstract

This study aimed to determine whether microneedles (MN) can enhance the efficacy of a commercially available topical anesthetic (EMLA®) when applied to the palatal mucosa. In a randomized, double-blind, crossover trial, 30 male volunteers received bilateral applications of MN patches and flat patches (FL, without MN) on the palatal mucosa. EMLA® was subsequently applied for either 2 or 5 min in separate sessions. Immediately thereafter, infiltrative anesthesia was administered, and pain intensity associated with needle insertion and local anesthetic injection was measured using a 0-100 Visual Analogue Scale. No differences in pain intensity were observed during needle insertion or local anesthetic injection when MN and FL patches were compared, irrespective of the duration of EMLA® application. However, a significantly higher proportion of participants reported pain-free infiltrative anesthesia when EMLA® was applied for 2 min following MN pretreatment (40%) compared with FL pretreatment (10%) (p = 0.008). MN application was not associated with adverse effects. Pretreatment with MN enhances the clinical efficacy of intraoral topical anesthesia in males. These findings open new avenues for exploration of advanced transbuccal drug delivery technologies focused on fast-release systems that may improve clinical outcomes in topical dental anesthesia.

## Introduction

Local anesthesia is administered with the aim of blocking nociceptive signaling and, ultimately, alleviating pain during dental procedures.^1^ Paradoxically, the procedure itself—needle insertion and injection of the local anesthetic solution—may induce pain and discomfort, which can lead to the development of aversion to dental treatment and, consequently, compromised oral health-related quality of life and reduced utilization of dental care services.^2^

In the pursuit of less painful local anesthesia, topical anesthetics have emerged as a valuable adjunct. These agents act by reducing tissue sensitivity, thereby alleviating patient discomfort not only during dental injections but also during procedures such as biopsies and minor soft-tissue surgical interventions. However, commercially available topical anesthetics do not consistently provide effective superficial anesthesia.^3,4^

Among the various regions of the oral cavity, the palatal mucosa poses particular challenges for achieving adequate topical anesthesia. This difficulty is primarily attributed to the keratinized epithelium, which limits drug absorption, and the firm adherence of the mucosa to the underlying bone.^5^ In addition, injection of the anesthetic solution into this area requires greater pressure than usual, potentially resulting in increased patient discomfort.^2^

To address these challenges, previous studies have explored extending the application time of topical anesthetics, for example, prolonging application to 5 min rather than the conventional 1–2 min. However, although this strategy has been shown to reduce pain associated with needle insertion, it has not been effective in completely eliminating pain during injection of the local anesthetic into the palatal region.^3,6^ Consequently, alternative strategies beyond prolonging application time are required to facilitate penetration and enhance the effectiveness of topical agents in this context.

In transdermal drug delivery, the use of microneedles (MN) has been shown to be an effective strategy for enhancing the efficacy of topical anesthetics.^7–9^ These studies demonstrated improved EMLA® effectiveness when applied for periods ranging from 20 to 50 min to skin pretreated with MN with a minimum height of 500 μm. Such pretreatment has been employed in various dermatological procedures, including carbon dioxide laser applications,^7^ treatment of acne scars,^8^ and photoelectric testing.^9^ This approach has likewise emerged as a promising method for enhancing the efficacy of topical formulations applied within the oral cavity.

For example, the use of 750-μm stainless-steel MN has been shown to be a minimally painful procedure compared with a 30G hypodermic needle when applied to the oral cavity of human volunteers.^10^ Moreover, since the oral mucosa is highly permeable and readily accessible for drug application aimed at systemic effects, MN offer the potential for significantly enhancing the efficacy of multiple drug categories. Indeed, MN have been employed for drug and vaccine delivery in the oral cavity, supporting their potential as an alternative approach to both oral and parenteral drug administration.^11,12^

More recently, a fast-dissolving lidocaine MN patch was shown to be safe and capable of effective retention when applied for 3 min to the tongues of rabbits, even in the presence of muscle and salivary movements. Notably, this patch exhibited superior *in vivo* efficacy compared with EMLA® and topical lidocaine 5%, as demonstrated using a tail-flick model in mice.^11^

In a clinical study involving 15 volunteers, 400-μm-long MN significantly enhanced the effectiveness of topical lidocaine 5% when applied to the palate and buccal mucosa. Concurrent application of MN and the topical anesthetic for 3 min resulted in a marked reduction in pain associated with needle insertion and subsequent local anesthetic injection, particularly when compared with the control site that received the topical anesthetic alone.^13^

Consequently, since MN have been shown to enhance absorption and efficacy of topical anesthetics when applied to pretreated skin, and effective topical dental anesthesia remains a significant clinical challenge, the present study aimed to assess the therapeutic potential of intraoral MN pretreatment for improving the anesthetic effect of EMLA®. Only limited literature has evaluated this approach in the context of the oral cavity. The hypothesis was that MN pretreatment would reduce pain intensity during needle insertion and local anesthetic injection. A secondary hypothesis was that extending the duration of topical anesthetic application would improve anesthetic efficacy.

## Methods

### Study design and ethical aspects

The Brazilian National Committee for Ethics in Research, National Health Council, Ministry of Health (CONEP/CNS/MS) approved this research (CAAE number: 39194814.8.0000.5418), and it was conducted in accordance with the Declaration of Helsinki. The study was registered on the ClinicalTrials.gov website (Identifier: NCT05267938). Reporting of this study was conducted in accordance with the Consolidated Standards of Reporting Trials (CONSORT) guidelines.

Male participants underwent anamnesis and a comprehensive clinical examination of the oral cavity. Individuals who met the inclusion and exclusion criteria were invited to participate in the study and were required to provide written informed consent (Resolution 466/12 of the Brazilian National Health Council).

The inclusion criteria consisted of healthy male individuals with excellent oral health, specifically undergraduate or graduate students at Piracicaba Dental School. The exclusion criteria included individuals identified as smokers or alcoholics, those who had taken medications known to affect pain perception (e.g., analgesics, anti-inflammatory drugs, antidepressants, or anxiolytics), or those who had received local anesthesia in the oral cavity within the two weeks preceding the study.

The efficacy of MN pretreatment on the palatal mucosa was evaluated in a randomized, crossover, split-mouth, double-blind study. Thirty healthy male volunteers participated, with a mean age (SD) of 26.3 (3.2) years. Among them, 29 (96.67%) self-identified as white, and 1 (3.33%) as of African descent. Participants underwent two separate sessions, and all clinical procedures were performed by the same trained dentist.

**Figure 1:**
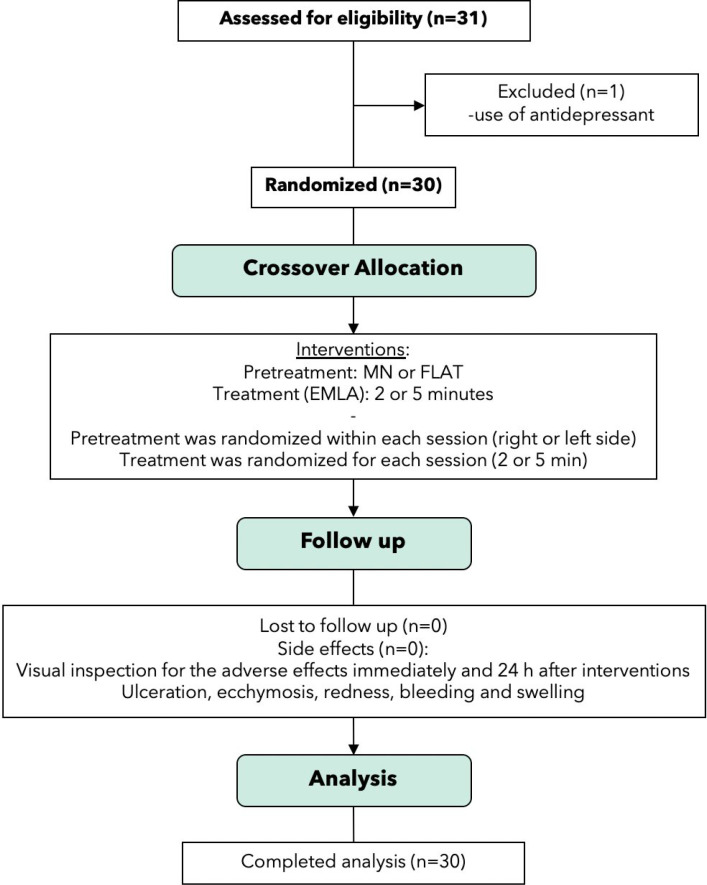
CONSORT diagram shows patient flow through this clinical study.

VAS: visual analogue scale; EMLA: eutectic mixture of local anesthetics; MN: microneedle patch; FL: flat patch (without microneedles).

On one side of the palate (1 cm from the gingival margin, between the first and second premolar regions), participants received the MN patch coupled to an application system. On the contralateral side, serving as the negative control, participants received a flat patch without MN (FL). After patch application, EMLA® (AspenPharma, Serra, Espírito Santo, Brazil) was applied for either 2 or 5 min before administration of infiltrative anesthesia using a 30G dental needle (Injex, Ourinhos, São Paulo, Brazil). Pain assessment was performed at two time points: during needle insertion and during injection of the anesthetic solution. Figure 1 illustrates the study design.

### MN patches

MN patches were prepared based on a method previously developed and described by Dr. Gill's research group.^14,15^ Briefly, an MN patch was designed using AutoCAD software (Autodesk, Cupertino, USA). The patch comprised 57 MN, each measuring 750 μm in length and 200 μm in width, fabricated from stainless steel 316 plates with a thickness of 50 μm. The stainless steel plates were manufactured using a wet-etching technique provided by Tech-Etch (Plymouth, USA). Subsequently, the MN were thoroughly rinsed in running water and dried using compressed air. Each microneedle was manually bent to a 90° angle under microscopic visualization. The negative control utilized the same microneedle patch; however, the MN were not bent and remained flat, thereby preventing tissue perforation.

### Application system

The application system utilized in this study was adapted from an applicator previously developed by our research group.^10^ The applicator consisted of a 5 mL syringe (Descarpack®, São Paulo, , Brazil; lot: SLLAA0014) equipped with a spring with an external diameter of 10 mm and a force constant of 2.55 N/mm. Spring deformation was controlled by depressing the plunger to a depth of 2 mm (equivalent to one syringe unit line), thereby regulating the force exerted by the application system.

**Figure 2:**
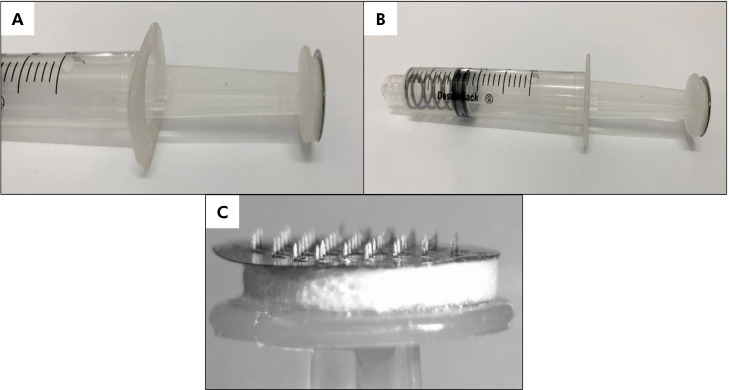
Images A and B represent the application system with the foam adaptation affixed to the flat face of the plunger. Figure C illustrates an approximate view of the system, showcasing the foam and the microneedles patch. This image was captured using a Stereo microscope with an integrated photographic camera (OPTIKA, mode SZX-T, Pontarlanica - Bergamo, Italy).

The application force was set at 10 N, a value previously calibrated and confirmed in accordance with prior work.^10^ To facilitate more comfortable application for the volunteers, given the challenging nature of the application site, an MN patch (either with MN bent at 90° or remaining flat) was securely affixed to the flat face of the plunger using foam material and adhesive tape (3M). Figure 2 shows the modified application system used in this study.

To confirm that the MN attached to the modified application system (with foam) effectively penetrated the palatal mucosa, particularly in areas with irregularities due to palatine ridges, a preliminary study was conducted. To assess the performance of the adapted applicator, 5 volunteers underwent application of MN patches using both systems in a randomized order. After MN removal, any micropores created in the mucosa were stained with 1% gentian violet. Subsequently, the stained micropores were photographed using a digital camera (Canon EOS XTI; Canon 100-mm macro lens; Canon MR-14EX II ring flash) and counted to quantify the degree of penetration.

### Screening and treatments

The study was divided into two separate clinical sessions, with a one-week interval between them. Several factors, including EMLA® application time (2 or 5 min), treatment side of the palate (right or left), and treatment type (MN or FL), were randomized in advance using a computer-generated list.

The dentist responsible for performing the applications received information regarding procedural details only at the start of each session, and the specific treatment allocation for that session remained concealed until the time of application. Table 1 illustrates the application sequence for two volunteers according to their respective randomization.

**Table 1:** Example of treatment randomization for two volunteers in which the EMLA® application time (2 or 5 min), the treatment side (left or right), and the treatment type (MN, microneedle patch; FL, flat patch) were independently randomized.

Variables		EMLA® application time	Application side	Treatment type
Volunteer 1				
	1^st^ session	2 min	Left	MN
			Right	FL
	2^nd^ session	5 min	Right	FL
			Left	MN
Volunteer 2				
	1^st^ session	5 min	Right	MN
			Left	FL
	2^nd^ session	2 min	Right	MN
			Left	FL

Participants were blindfolded before treatment to maintain study blinding. In addition, the dentist responsible for pain assessment and the statistician involved in data analysis were kept unaware of the treatment allocation for each participant. All applications were performed by the same dentist in both sessions, thus ensuring procedural consistency.

**Figure 3:**
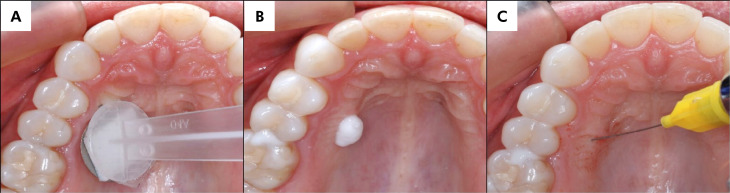
Images A, B, and C provide a visual representation of the sequential application of treatments in this clinical study, as viewed indirectly through the use of a mirror: (A) MN application using the application system; (B) the application of the topical anesthetic, and (C) demonstrates the procedure for administering local anesthesia.

Before treatment, the application site was dried using gauze. Treatments involving MN or FL patches were performed on the palatal surface, targeting the area between the first and second premolars, approximately 1 cm from the teeth (Figure 3A), according to the predetermined computer-generated randomization order.

Immediately after patch application, 100 mg of the topical anesthetic formulation (EMLA®), previously weighed according to established protocols^3,16^, was applied to the pretreated area (Figure 3B). The formulation was then removed with gauze, and infiltrative anesthesia was administered using a 30G dental needle and 0.3 mL of 2% lidocaine with epinephrine 1:100,000 (Alphacaine®, DFL, Rio de Janeiro, Brazil) (Figure 3C).

Participants were instructed to assess pain at two time points: during needle insertion until contact with the periosteum and during injection of the anesthetic solution. The procedure was then repeated on the contralateral side of the palatal mucosa.

Pain associated with each treatment was assessed using individual Visual Analogue Scales (VAS). Each scale consisted of a 100-mm horizontal line without demarcations, anchored by the descriptors "no pain" (left end) and "unbearable pain" (right end).^17^ Participants were asked to mark the scale vertically to indicate perceived pain intensity at each procedure stage. Subsequently, a blinded researcher measured the distance, in millimeters, from the left anchor ("no pain") to the participant's mark to quantify pain intensity.

### Follow-up

Immediately after the interventions and again 24 hours later, the application sites underwent visual inspection to assess the occurrence of adverse effects. These effects included, but were not limited to, bleeding, ulceration, ecchymosis (bruising), redness, and swelling.

### Statistical analysis

The following parameters were considered for sample size calculation: an effect size *f* of 0.25 for differences in pain intensity during needle insertion and anesthetic injection between MN and FL patches in an analysis of variance (ANOVA) model with two within-subject factors, a correlation between repeated measures of at least 0.45, a power of 80%, a significance level of 5%, and an anticipated dropout rate of approximately 10%. Based on these assumptions, the required sample size was 30 participants.

Pain intensities associated with needle insertion and injection were reported as the median and interquartile range (IQR), unless otherwise specified. Normality was assessed using the Kolmogorov–Smirnov test, and log_10_ transformations were applied to the pain intensity values when necessary, with a significance level of 5% (*p* < 0.05).

A repeated-measures two-way ANOVA was performed to evaluate differences in pain intensity following needle insertion and injection (using log_10_-transformed values). The within-subject factors were application time (two levels: 2 and 5 min) and pretreatment (two levels: MN and FL). When appropriate, multiple comparisons were conducted using Tukey's honestly significant difference (HSD) test, with the significance level set at 5% (p = 0.05).

Cochran's Q test was used to compare the proportions of pain-free local anesthesia procedures (VAS < 4 mm^18^) for needle insertion and injection across combinations of application time and pretreatment. When required, post hoc analyses were performed using Dunn's test, with the significance level set at 5% (p = 0.05).

## Results

### Confirmation of MN penetration into the palatal tissue

The preliminary study demonstrated that the foam-enabled applicator perforated the palatal tissue to a similar extent as the original applicator (mean ± SD for MN and MN + foam: 46.8 ± 6.0 and 39.0 ± 9.7, respectively). These preliminary data indicate the feasibility of this system.

**Figure 4:**
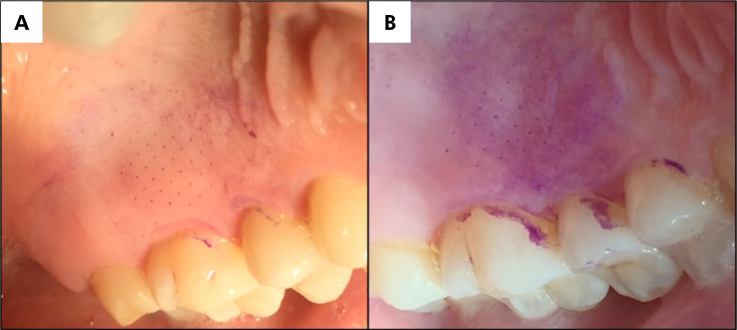
Images A and B were captured immediately after MN insertion and the subsequent application of gentian violet 1% to highlight the micropores. (A) depicts the application system with the new foam adaptation, and (B) represents the original application system.

Figure 4 provides a visual comparison between the micropores created by the foam-adapted application system (A) and those created by the original system (B).

### Pain following needle insertion and injection

**Figure 5:**
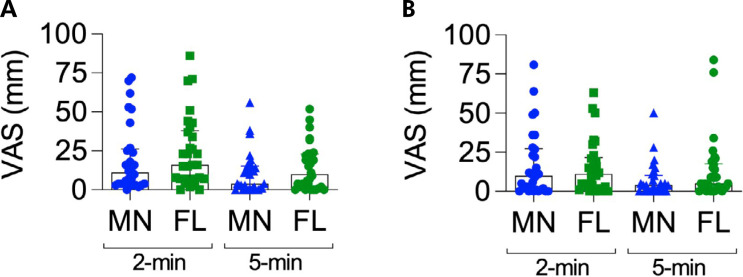
Median (interquartile deviation) of pain intensity assessed through VAS (in mm) after needle insertion (A) and local anesthetic injection (B) in the palate pretreated with microneedles patch (MN) and negative control (FL), after 2 or 5 min application of EMLA^®^. Repeated-measures two-way ANOVA, followed by the Tukey test. (n = 30).

Figure 5 shows pain intensity following needle insertion and local anesthetic injection in the palatal region. Overall, MN pretreatment of the palatal region did not result in a significant reduction in pain intensity during needle insertion, irrespective of EMLA® application time (F_1,29_ = 2.07, p = 0.160 and partial η^2^ = 0.06, Figure 5A). However, a significant main effect of time was observed, with the 5-min application of EMLA® being more effective in reducing pain intensity during needle insertion than the 2-min application (F_1,29_ = 6.09, *p* < 0.001, and partial η^2^ = 0.33, Figure 5A).

Similarly, MN pretreatment of the palatal region did not lead to a significant reduction in pain intensity during local anesthetic injection, regardless of EMLA® application time (F_1,29_ = 2.05, p = 0.162, partial η^2^ = 0.06; Figure 5B). Nevertheless, a significant main effect of time was observed, with the 5-min application of EMLA® being more effective than the 2-min application in reducing pain intensity during local anesthetic injection (F_1,29_ = 7.34, p = 0.011 and partial η^2^ = 0.20; Figure 5B).

Regarding the proportions of pain-free local anesthesia procedures (VAS < 4 mm during both needle insertion and injection), significant differences were observed (χ^2^ = 16.5, and p = 0.001, Table 2). Pairwise comparisons indicated that the proportion of participants reporting pain-free needle insertion and injection after 2-min application of EMLA® and MN pretreatment (40%) was significantly higher than after 2-min application with FL pretreatment (10%) (p = 0.008). However, no differences were observed between the pretreatments after 5-min application of EMLA® (44% for MN and 24% for FL, p > 0.05).

**Table 2:** Proportions and 95% confidence intervals (CI) for participants who reported pain-free local anesthesia (< 4 mm on the Visual Analogue Scale during both needle insertion and injection) following EMLA® application for 2 and 5 min and pretreated with either microneedle (MN) or a flat patch (FL).

Pretreatment	EMLA® Application time	Pain-free needle anesthesia	
		n (%)	95%CI
MN	2 min	12 (40)^a^	22– 57
	5 min	13 (44)^A^	25–61
FL	2 min	3 (10)^b^	0–20
	5 min	7 (24)^A^	8–38

Different lowercase letters indicate a significant difference for the 2-min application of EMLA®. Same uppercase letters indicate no significant difference for the 5-min application of EMLA®.

### Incidence of adverse effects after MN application in the palate

No volunteer exhibited adverse reactions, such as ulceration, ecchymosis, redness, bleeding, or swelling, following application of MN or the negative control, either immediately after treatment or 24 hours later, demonstrating the safety of MN use in the palatal region. Furthermore, the absence of participant withdrawal indicates that the treatments were well tolerated and accepted by all participants, reasserting the overall safety and acceptability of the study procedures.

## Discussion

This study confirmed the hypothesis that MN pretreatment enhances the efficacy of a 2-min application of a topical anesthetic when administered intraorally in male participants, resulting in a significantly higher proportion of pain-free local anesthesia with a commercially available topical anesthetic (EMLA®). However, when application duration was extended, MN pretreatment did not influence the efficacy of EMLA®.

Notably, despite differences in permeability and histological characteristics between application sites, several clinical trials have reported that application times ranging from 10 to 30 min for EMLA® on MN-pretreated skin significantly enhance anesthetic efficacy.^7–9,19,20^ These studies corroborate the findings of the present study, demonstrating that MN pretreatment enhances the delivery and efficacy of topical anesthetics. While longer application times are required to achieve effective anesthesia in less permeable tissues such as skin, the shorter durations observed in this study were sufficient for intraoral mucosa—a more permeable site—particularly when MN pretreatment was employed.

Similarly, a previous study demonstrated that an MN patch combined with topical lidocaine 5%, applied in parallel for 3 min, reduced pain during needle insertion, periosteum contact, and local anesthetic injection in the buccal and palatal mucosa of 15 volunteers.^13^ Despite methodological differences between studies, including variations in topical formulation, application method, MN size and shape, and amount of topical anesthetic used (500 mg vs 100 mg), the findings consistently highlight the potential of MN application to enhance the efficacy of intraoral topical anesthesia.

The choice of EMLA® application times in the present study aligns with typical practices in dental clinical trials and clinical settings, in which application durations range from 30 s to 5 min.^21,22^ Notably, previous studies have shown that EMLA® can induce a hypoalgesic effect in the gingival mucosa of the lower incisors after 2, 5, or 10 min of application.^23^ However, the clinical feasibility of prolonged topical application times must be considered, since such durations may be impractical for routine dental practice. Additionally, prolonged application of topical formulations may increase the risk of toxicity. For example, prolonged EMLA® application (for 30 min) has been reported to cause oral mucosal ulceration and desquamation in the upper right lateral incisor region.^24^

Interestingly, extending the duration of topical anesthetic application from 2 to 5 min in the MN-pretreated area provided no additional benefit over the efficacy of EMLA®. This finding suggests that local anesthetic permeation after a 5-min application is similar regardless of the presence of microperforations. Moreover, this observation indicates that MN pretreatment can shorten application times for topical anesthetics and enhance the cost–benefit of the procedure. Notably, this outcome may be attributed to the established clinical efficacy of EMLA®, underscoring its selection as the topical anesthetic of choice for maximizing anesthetic effects.^4^ Previous studies have consistently demonstrated the superior performance of EMLA® in the oral cavity compared with other commercially available formulations, such as benzocaine or lidocaine alone.^6,16,25,26^ Furthermore, *in vitro* studies have demonstrated EMLA®'s ability to permeate the palatal mucosa epithelium^16^ and full-thickness palatal mucosa pretreated with MN, both evaluated using Franz-type vertical diffusion cells.^27^ These findings collectively support the selection of EMLA® as the topical anesthetic for the present study, given its established clinical efficacy and ability to penetrate palatal tissues.

Microneedles have emerged as a promising technology for enhancing transdermal drug delivery, demonstrating efficacy across multiple drug classes. They are manufactured from a wide variety of materials, and their designs vary in size and geometry.^28,29^ More recently, their applications have been extended to transbuccal drug delivery, highlighting their potential for oral mucosal drug administration.^30^ In the present study, 750-μm-long MN were selected to penetrate the thick keratinized stratified squamous epithelium (approximately 500 μm thick), and the transverse palatal ridges (rugae), which constitute a primary barrier to permeation of topically applied drugs in the oral cavity.^5^ Similarly, previous research has shown that MN patches of the same size and material successfully penetrate various oral cavity regions, including the palate, with lower pain perception compared with a 30G hypodermic needle.^10^ Furthermore, MN longer than 1500 μm have been reported to elicit pain perception similar to that of 26G hypodermic needles when applied to the skin of the forearm, suggesting that longer MN may be unsuitable for intraoral use.^31^

The VAS is widely used to assess pain intensity in clinical trials evaluating intraoral topical anesthetics or discomfort during needle penetration procedures.^32–36^ Nevertheless, the intangible and subjective nature of pain perception can make detection of small differences in pain levels challenging, particularly in studies with relatively small sample sizes and in which mild pain intensity—i.e., VAS scores below 40 mm—is expected for most participants,^18,37^ as was the case in this study. Consequently, it is not surprising that MN application did not differ significantly from FL patches regarding pain intensity during needle puncture or local anesthetic injection. Furthermore, interindividual variability and the lack of consensus regarding analysis and interpretation of VAS-based pain reports^17,38^ may pose further challenges for translating research findings into clinical practice.

Given the potential advantages of MN as an advanced drug delivery system, the most clinically relevant outcome is arguably whether local anesthesia is minimally painful or entirely pain-free. Therefore, consistent with previous findings^39^, evaluating the proportion of patients achieving a pain-free procedure provides a more meaningful perspective on the benefits of MN pretreatment. Moreover, considering needle insertion and anesthetic injection as a composite procedure may better reflect clinical practice, since both steps are integral to achieving effective local anesthesia. To this end, a VAS score of < 4 mm, as proposed by Jensen and colleagues,^18^ was adopted as the threshold for defining successful pain-free local anesthesia. The results showed that 40% of volunteers reported pain-free local anesthesia after MN pretreatment of the palatal mucosa with a 2-min application of EMLA®. This finding is particularly noteworthy, representing a 30% increase in efficacy compared with topical anesthetic application using FL patches for the same duration, and is comparable to the incidence of pain-free local anesthesia observed after MN pretreatment with a 5-min application. These results highlight the potential clinical utility of MN technology for improving the effectiveness of intraoral anesthesia.

This study has some limitations related to the demographic characteristics of the sample. Pain intensity was assessed without potential bias related to the menstrual cycle, which is known to influence pain perception.^40^ Consequently, the findings cannot be extrapolated to women or other populations, such as older adults or children. Nevertheless, these groups represent important targets for future research aimed at enhancing the anesthetic effects of topical anesthesia, particularly in pediatric dentistry, where minimizing challenges associated with local anesthesia is of critical importance.

## Conclusion

This study demonstrated a significant improvement in the effectiveness of EMLA® when applied to palatal mucosa pretreated with 750-μm-long stainless steel microneedles in male participants. The findings encourage further exploration of novel transbuccal drug delivery technologies focused on fast-release systems, which may offer improvements in the effectiveness of topical anesthesia.

## Data Availability

The datasets generated during and/or analyzed during the current study are available from the corresponding author on reasonable request.
